# Chronic Myeloid Leukemia as a Secondary Malignancy Following Treatment of Diffuse Large B-Cell Lymphoma

**DOI:** 10.4274/Tjh.2013.0180

**Published:** 2014-03-05

**Authors:** Itır Şirinoğlu Demiriz, Emre Tekgündüz, Sinem Civriz Bozdağ, Fevzi Altuntaş

**Affiliations:** 1 Ankara Oncology Hospital, Department of Hematology and Stem Cell Transplantation Unit, Ankara, Turkey; 2 Ankara Oncology Education and Research Hospital, Ankara, Turkey

**Keywords:** Lymphoma, Chronic myeloid leukemia, Secondary malignancy

## TO THE EDITOR

Philadelphia (Ph) chromosome (t(9; 22)(q34; q11))-positive chronic myeloid leukemia (CML) occurring as a secondary malignancy in patients who were treated for non-Hodgkin lymphoma (NHL) is very rare [1,2]. The association between B-cell–derived lymphoid neoplasias and myeloproliferative disorders is not clear [1]. Until now, CML has been reported after treatment for Hodgkin disease (HD), hairy cell leukemia, or chronic lymphocytic leukemia (CLL). It is not clear whether development of CML as a secondary malignancy represents a therapy-induced complication or possibly a genetic susceptibility to malignancy in which the host may be able to bear 2 different clonal malignanT-cells [3,4]. There is also a possibility that the 2 malignant clones derive from a common malignant stem cell [4]. 

A 45-year-old female was admitted to our hospital in July 2006. An undifferentiated malignant tumor was detected following upper gastrointestinal system endoscopy. Biopsy revealed a high-grade, CD20-positive malignant lymphoma. The general surgery department performed a near-total gastrectomy for the mass lesion, of 8x6 cm in size. She was referred to our hematology clinic with a diagnosis of diffuse large B-cell lymphoma (DLBCL). Computerized tomography scans, bone marrow aspiration, and biopsy results revealed Ann Arbor stage IEB disease with a normal karyotype, and fluorescence in situ hybridization (FISH) analysis results were negative for t(8,14) and t(14,18). Informed consent was obtained.

Six courses of R-CHOP chemotherapy were completed in December 2006 with achievement of complete remission (CR). She was followed in CR until December 2010, at which point the patient presented with leukocytosis and thrombocytosis (white blood cell count: 61.5x10^9^/L, neutrophils: 5.1x10^9^/L, hemoglobin: 12.7 gr/dL,platelet count: 754x10^9^/L). Physical examination was normal. Peripheral blood smear showed leukoerythroblastosis and mild basophilia with 32% metamyelocytes and 21% myelocytes. Bone marrow aspiration and biopsy revealed hypercellular bone marrow and myeloid hyperplasia (M/E: 6/1), and 1.4% basophilia, but no blastic infiltration or fibrosis. FISH analysis showed 93% Ph chromosome positivity, whereas JAK-2 mutation was not detected. She was diagnosed with CML in the chronic phase. Her Sokal score was 0.73 (low) and imatinib mesylate therapy at 400 mg/day was initiated in January 2011. Complete hematological response was achieved in March 2011, followed by complete cytogenetic and major molecular responses in June 2011. She is still being followed in our outpatient clinic with major molecular response.

Primary DLBCLs are aggressive tumors accounting for approximately 40% of all B-cell malignancies. Up to 40% of the masses are extranodal. The most common extranodal site is the stomach. Patients who have been treated for NHL have an increased risk of developing secondary malignancies, including acute myeloid leukemia, CLL, and solid tumors of various types. 

Epidemiologic studies performed in large series have shown the possibility of secondary CML occurrence in cases of HD, NHL, and various solid tumors [5,6,7,8,9]. Usually, the median latency of time between the 2 diseases was 60 months, and the majority of the patients were diagnosed with CML in the chronic phase. Secondary CML characteristics were similar to those of de novo cases in a series by Bauduer et al. [5]; however, another report suggested a lower incidence of splenomegaly and hyperleukocytosis associated with therapy-related CML. 

Whang-Peng et al. [10] reported secondary leukemia in patients with different types of malignancies. CML was observed in 8 patients. In 1 patient, CML developed after treatment for NHL. The remaining patients had cancer of the breast, CLL, HD, or acute lymphoblastic leukemia. 

Several explanations appear possible for the occurrence of CML in DLBCL. CML is a disease of the pluripotent stem cells that involves not only myeloid but also lymphoid cell compartments. First, the cytostatic drugs used in treatment of DLBCL may be directly involved in the pathogenesis of CML. Second, therapy-induced immune dysregulation, such as radiotherapy, may contribute to the evolution of CML. Third, theoretically but not yet proven, the neoplastic transformation of a progenitor cell, capable of differentiation into either lymphoid or myeloid cell lines, might lead to the association of lymphoproliferative and myeloproliferative disorders. Therefore, it might have been likely that the Bcr-abl rearrangement was present in the DLBCL cells [2].

 Secondary CML patients receiving treatment for NHL that have been reported so far are presented in Table 1. In all patients, prior chemotherapy and/or radiotherapy and latency time between the 2 diseases strongly suggested the secondary nature of CML [7,10,11,12,13,14].

On the other hand, this case report implies that patients treated for NHL require close follow-up for years after completing therapy. In conclusion, patients with lymphoproliferative malignancies like DLBCL can present many years later after successful treatment of their disease with a clonal myeloproliferative disease like CML. Molecularly targeted therapy with tyrosine kinase inhibitors seems to be effective and well tolerated in patients with secondary CML.

## CONFLICT OF INTEREST STATEMENT

The authors of this paper have no conflicts of interest, including specific financial interests, relationships, and/or affiliations relevant to the subject matter or materials included.

## Figures and Tables

**Table 1:**
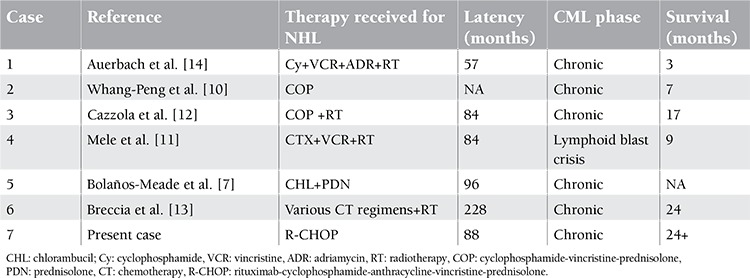
Reported cases of CML developing after NHL.
